# Fisheries-induced neutral and adaptive evolution in exploited fish populations and consequences for their adaptive potential

**DOI:** 10.1111/eva.12220

**Published:** 2014-12-02

**Authors:** Lise Marty, Ulf Dieckmann, Bruno Ernande

**Affiliations:** 1 IFREMER, Laboratoire Ressources Halieutiques, Unité Halieutique Manche-Mer du NordBoulogne-sur-mer, France; 2 IIASA, Evolution and Ecology ProgramLaxenburg, Austria

**Keywords:** eco-genetic model, effective population size, fisheries-induced evolution, genetic drift, genetic erosion, genetic markers, life-history traits, natural selection and contemporary evolution

## Abstract

Fishing may induce neutral and adaptive evolution affecting life-history traits, and molecular evidence has shown that neutral genetic diversity has declined in some exploited populations. Here, we theoretically study the interplay between neutral and adaptive evolution caused by fishing. An individual-based eco-genetic model is devised that includes neutral and functional loci in a realistic ecological setting. In line with theoretical expectations, we find that fishing induces evolution towards slow growth, early maturation at small size and higher reproductive investment. We show, first, that the choice of genetic model (based on either quantitative genetics or gametic inheritance) influences the evolutionary recovery of traits after fishing ceases. Second, we analyse the influence of three factors possibly involved in the lack of evolutionary recovery: the strength of selection, the effect of genetic drift and the loss of adaptive potential. We find that evolutionary recovery is hampered by an association of weak selection differentials with reduced additive genetic variances. Third, the contribution of fisheries-induced selection to the erosion of functional genetic diversity clearly dominates that of genetic drift only for the traits related to maturation. Together, our results highlight the importance of taking into account population genetic variability in predictions of eco-evolutionary dynamics.

## Introduction

Anthropogenic activities do not only affect population dynamics in the wild, but can also have evolutionary consequences. Harvesting, habitat fragmentation, pollution and many other pressures affect the genetic composition of populations through selection for certain trait values or through altered rates of genetic drift (Allendorf et al. 2008; Hendry et al. 2011). Selection and genetic drift interact via their effects on standing genetic variation, and together determine evolutionary dynamics.

Harvesting may increase genetic drift, because it reduces population size and alters population structure in age, size and maturity status; it may also modify sex ratio, as in trophy hunting in which males are selectively targeted (e.g. in ungulates; Martínez et al. 2002). All these effects may reduce the effective population size *N*_e_ (Wright 1938), which, in the absence of sources of new alleles (migration or mutation), may deplete genetic variability, a process known as genetic erosion. Unlike most genes coding for quantitative traits, which are still in the process of being identified by modern genomic methods, neutral molecular markers have been increasingly used over the past decades to aid the conservation of natural populations (e.g. Palsbøll et al. 2007). Regarding fish stocks, they have allowed investigating the historical influences of past fishing pressures on neutral genetic diversity, using DNA extracted from archived otoliths or scales. Some of these studies, mostly based on microsatellites and/or mitochondrial DNA, found a loss of neutral genetic diversity (Hauser et al. 2002; Hutchinson et al. 2003; Hoarau et al. 2005), while other did not (Ruzzante et al. 2001; Therkildsen et al. 2010). This disparity could originate in the low numbers of individuals and/or loci sampled, resulting in inaccurate *N*_e_ estimates due to sampling error in measuring population allele frequencies (Waples 1998). In marine species, bias can also be caused by high gene flow (Wang and Whitlock 2003). However, a recent cross-species analysis comparing neutral genetic diversity in harvested and nonharvest fish stocks has found it to be lower in the former case than in the latter (Pinsky and Palumbi 2014).

Also functional genetic diversity is theoretically susceptible to erosion through genetic drift. Without selection, and when *N*_e_ becomes very small (a few tens), additive genetic variance is, along with neutral genetic variance, expected to increase logistically with effective population size, because of weaker drift and an increased number of mutations (Willi et al. 2006). However, a loss of additive genetic variation due to genetic drift in harvested fish stocks has not been empirically documented, and is not expected according to current knowledge on effective population sizes in marine fish, which are several orders higher than values required for this to happen.

In contrast, harvest-induced genetic changes through the adaptive evolution of life-history traits have been reported in a number terrestrial species (e.g. Jachmann et al. 1995; Coltman et al. 2003), as well as for harvested fish (see reviews by Jørgensen et al. 2007; Kuparinen and Merilä 2007; Hutchings and Fraser 2008). According to life-history theory, high levels of fishing mortality and size-selectivity favour individuals with slow growth, early maturation at small size and high reproductive investment – predictions that have been empirically corroborated (reviewed in Jørgensen et al. 2007). These phenotypic changes can undermine a stock's renewal capacity if the reduction in fecundity due to smaller adult body size is higher than the gain in fecundity due to higher reproductive investment and may become maladaptive once fishing pressure is released. Besides affecting average phenotypes, fisheries-induced selection may also affect genetic diversity in quantitative traits. After a directional episode, selection can turn stabilizing and, if not counterbalanced by gene flow or high mutation rates, may reduce additive genetic variance. The latter determines not only the rate at which characters respond to selection, but also the scope of this response in the short term.

Eco-genetic models have been used to explore the eco-evolutionary consequences of harvesting on fish populations (e.g. Baskett et al. 2005; Dunlop et al. 2007, 2009a,b; Thériault et al. 2008; Enberg et al. 2009). Previous studies found, in particular, that fisheries-induced genetic adaptations were reversed once fishing was stopped but that the evolutionary reversal rate was much slower than the fisheries-induced evolutionary rate, because natural selection pressures were weaker than fisheries-induced ones. However, the influence of fishing on the amount of additive genetic variance and its consequences for the reversal of fisheries-induced adaptations have not yet been investigated. This is because previous studies investigated trait evolution using the infinitesimal quantitative genetic model that describes traits as affected by an infinite number of loci, which precludes any significant loss of additive genetic variation (but see Wang and Höök 2009; Kuparinen and Hutchings 2012). In addition, the response to selection also depends on effective population size, because, in small populations, genetic drift is stronger and can counteract selection.

Here, we develop an individual-based eco-genetic model (Dunlop et al. 2009b) with gametic inheritance of traits coded by finite numbers of loci and alleles per locus, so that some alleles can be lost due to selection and/or drift. We address the evolutionary dynamics at neutral loci, only affected by genetic drift, and at functional loci coding for several life-history traits, on which both genetic drift and selection act. Resulting population genetic and demographic properties are examined. We specifically tackle three questions: (i) Are the fisheries-induced evolutionary dynamics of life-history traits and their reversal during a subsequent moratorium different when traits are explicitly described by finite numbers of loci and alleles?; (ii) What is the relative importance of reduced selection strength, reduced additive genetic variance, and reduced effective population size on the timescale of the evolutionary recovery of life-history traits?; and (iii) What are the relative contributions of selection and genetic drift on fisheries-induced changes in additive genetic variance?

## Model description

We model individuals that are diploid hermaphrodites (i.e. we do not distinguish between males and females, albeit we base our model on female life history). Their genotypes comprise a set of neutral loci, to study neutral evolution, and a set of functional loci, to investigate the combination of neutral and adaptive evolution of life-history traits. Functional loci code for the life-history traits of individuals and thus, together with plastic responses of traits to environmental variation, affect the life-history processes of growth, maturation, reproduction and mortality, which jointly determine population dynamics. Ultimately, individual fitness, controlling the production of offspring to which genetic material is transmitted, is determined by an individual's functional loci and environment. The latter is altered by fishing, the emerging population dynamics through density dependence, and environmental stochasticity affecting resources are available for growth and recruitment.

Below we describe in turn the genetics of neutral markers and life-history traits, phenotypic expression, the associated individual-level life-history processes from which population dynamics emerge, and the measures of population genetic diversity used to monitor evolutionary dynamics (see Table1 for variable definitions and Table2 for parameter values).

**Table 1:** Model variables

	Variable	Symbol	Unit	Equations
Genotypic values	Alleles ( 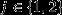 ) at locus *k* coding for trait *x*	*z*_*x*,*k*,*j*_(*i*)	–	1a
	Allelic value of allele *z*_*x*,*k*,*j*_(*i*)	*A*_*x*,*k*,*j*_(*i*)	See traits	1a,b
	Genotypic value of trait *x*	*A*_*x*_(*i*)	See traits	1b,2a,b
Phenotypic traits	Growth coefficient	*g*(*i*,*t*)	cm year^−1^	2a,b, 3b,c, 6b
	Growth investment at maturation onset	*α*(*i*)	–	2a, 3a
	Annual ratio of decay in postmaturation growth investment	*χ*(*i*)	–	2a, 3a
	PMRN intercept	*y*(*i*)	cm	2a
	PMRN slope	*s*(*i*)	cm year^−1^	2a
Emerging traits and individual state	Age	*a*(*i,t*)	Year	3a
	Age at maturation	*a*_m_(*i*)	Year	3a
	Fraction of productive season allocated to growth	*p*(*i*,*t*)	–	3a,b,c
	Somatic length	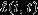	cm	3b,c, 4a, 6a, 7a
	Somatic weight	*w*(*i*,*t*)	g	
	Gonadic weight	*G*(*i*,*t*)	g	3c
	GSI	*Γ*(*i*,*t*)	–	
	Fecundity	*Q*(*i*,*t*)	–	5
	Maturation probability	*m*(*i*,*t*)	–	4a
	Length at 50% maturation probability	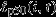	cm	4a
Population	Population biomass	*B*(*t*)	g	2b
	Number of recruits	*N*_0_(*t*)	–	5
	Mean offspring number per year at age *a*	*b*_*a*_(*t*)	–	
Mortality	Instantaneous size-dependent predation mortality rate	*d*_s_(*i*,*t*)	–	6a, 8
	Instantaneous growth-dependent mortality rate	*d*_g_(*i*,*t*)	–	6b, 8
	Size-selectivity function of fishery	*f*_trawl_(*i*,*t*)	–	7a,b
	Instantaneous harvest mortality rate	*d*_F_(*i*,*t*)		7b,8
	Total instantaneous mortality rate	*Z*(*i*,*t*)		8
	Death probability	*D*(*i*,*t*)		
	Survival probability until age *a*	*λ*_*a*_	–	
Population genetic diversity	Frequency of allele *l* at locus *k*	Π_k,l_(t)	–	9a,b, 11a,b
	Standardized variance in allele frequency change	*F*	–	9a,b
	Effective population size	*N*_e_	–	9b
	Generation time	*T*	Year	9b
	Additive genetic variance of trait *x*	*V*_A_(*x*)	trait unit^2^	10
	Phenotypic variance of trait *x*	*V*_P_(*x*)	trait unit^2^	10
	Heritability of trait *x*	*h*^2^(*x*)	–	10
	Expected heterozygosity	*H*_e_(*t*)	–	11a,b

–, dimensionless variable.

**Table 2:** Model parameters

	Parameter	Symbol	Value	Unit	Equations	Source
Genome structure	Number of neutral loci	*K*_n_	30	–	9a, 11a	
	Number of alleles per neutral locus	*L*_n_	10	–	9a, 11a	(1)
	Number of functional loci	*K*_f_	8	–	1a, 11b	
	Number of alleles per functional locus	*L*_f_	10	–	1a, 11b	(2)
Initial ranges of genotypic values	Growth coefficient	[*A*_*g*,min_, *A*_*g*,max_]	[6.0, 22.0]	cm year^−1^	1a	(3)
	Growth investment at maturation onset	[*A*_*α*,min_, *A*_*α*,max_]	[0.4, 1.0]	–	1a	(3)
	Rate of decay in postmaturation growth investment	[*A*_*χ*,min_, *A*_*χ*,max_]	[0.1, 0.5]	–	1a	(3)
	PMRN intercept	[*A*_*y*,min_, *A*_*y*,max_]	[40.0, 90.0]	cm	1a	(3)
	PMRN slope	[*A*_*s*,min_, *A*_*s*,max_]	[−1.0, 1.0]	cm year^−1^	1a	(3)
Expression noise	Noise coefficient of growth coefficient	*ɛ*_*g*_(*i*)	1 (1.19)*	–	2a,b	(4)
	Noise coefficient of growth investment at maturation onset	*ɛ*_*α*_(*i*)	1 (0.05)*	–	2a	(4)
	Noise coefficient of annual ratio of decay in postmaturation growth investment	*ɛ*_*χ*_(*i*)	1 (0.05)*	–	2a	(4)
	Noise coefficient of PMRN slope	*ɛ*_*y*_(*i*)	1 (0.24)*	–	2a	(4)
	Noise coefficient of PMRN intercept	*ɛ*_*s*_(*i*)	1 (6.7)*	–	2a	(4)
Growth	Strength of density dependence in growth	*ρ*	3 × 10^−9^	g^−1^	2b	(3)
	Production exponent	*β*	2/3	–		(3)
	Constant in allometric weight–length relationship	Ω	0.01	g cm^−3^	3c	(5)
	Initial length	ℓ_0_	10	cm		(3)
Maturation	PMRN envelope width	*ω*	20	cm	4b	(3)
	Lower bound of PMRN envelope	*p*_low_	0.25	–	4b	(6)
	Upper bound of PMRN envelope	*p*_up_	0.75	–	4b	(6)
Reproduction	Ratio of somatic to gonadic wet-weight energy densities	*γ*	0.62	–	3c	(3)
	Weight of an egg	*w*_egg_	4 × 10^−4^	g		(7)
	Maximum survival probability of recruits	*η*_1_	22 × 10^−7^	–	5	(7)
	Strength of density dependence in recruitment	*η*_2_	23 × 10^−12^	−	5	(7)
	Noise coefficient of recruitment	*ɛ*_*R*_(*t*)	1 (0.1)*	–	5	(7)
Natural mortality	Size-independent instantaneous natural mortality rate	*d*_0_	0.2	year^−1^	8	(3)
	Maximum instantaneous predation mortality rate	*c*_s_	0.6	year^−1^	6a	(3)
	Scaling factor of predation mortality rate	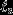	14	cm	6a	(3)
	Minimum instantaneous growth-dependent mortality rate	*c*_g_	0.02	year^−1^	6b	(3)
	Scaling factor of growth-dependent mortality rate	*g*_0_	6	cm year^−1^	6b	(3)
Fishing mortality	Steepness of the fishery's size-selectivity curve	*θ*	0.2	cm^−1^	7a	(7)
	Length at 50% selectivity	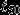	60	cm	7a	(8)
	Maximum instantaneous harvest rate	*H*	[0.2, 1.0]	year^−1^	7b	

−, dimensionless parameters.

(1) Poulsen et al. (2006); (2) by analogy with (1); (3) values chosen such that the life-history characteristics resemble those of North Sea cod (e.g. Marty et al. 2014); (4) standard deviation for each trait is determined such that the total expressed variance 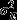 is related to: (i) an assumed initial additive genetic variance 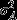 determined by an assumed initial genetic coefficient of variation *CV*_g_ of 6% and the initial mean trait values, and (ii) an assumed initial heritability *h*^2^ of 0.2, as 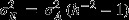; (5) values obtained from http://www.fishbase.org; (6) definition of PMRN width based on quartiles; (7) values taken from Enberg et al. (2009) and slightly modified when necessary; (8) between EU minimum landing size (35 cm) and asymptotic body size.

* Mean (standard deviation).

### Individual genotypes and phenotypes

#### Genetic inheritance

For both neutral and functional loci, genetic inheritance is modelled according to Mendelian laws under sexual reproduction: haploid gametes are formed for mature individuals by randomly drawing one of the two alleles at each locus, representing allelic segregation during meiosis. This is carried out independently between loci, so alleles can recombine freely, that is linkage is not included. Reproduction occurs between pairs of mature individuals (see section Reproduction for details) and the fusion of two randomly picked gametes creates a diploid offspring.

#### Neutral markers

To assess genetic drift, each individual carries 30 neutral loci. Genetic diversity at each neutral locus is represented by 10 possible allelic states distributed in the population, which mimics allelic diversity at microsatellite markers (Poulsen et al. 2006, for instance, reported a mean allelic diversity across loci and populations of 9.4 for cod).

#### Functional loci and genotypic values of life-history traits

Individuals have five evolving life-history traits, namely the juvenile growth rate *g*; two traits that specify the maturation schedule, that is the slope *s* and the intercept *y* of a linear probabilistic maturation reaction norm (PMRN; Heino et al. 2002a); and two parameters related to energy allocation between growth and reproduction after maturation, that is the proportion *α* of energy devoted to somatic growth in the first adult year and the annual ratio *χ* at which this proportion decreases throughout adult life (Table1; see section Life-history processes for more details).

For each individual *i*, the genotypic value of each trait results from *K*_f_ diploid functional loci. The two alleles at each locus can take *L*_f_ different possible allelic states. For a given trait, functional alleles act additively at and between loci. Dominance between alleles and epistasis between loci is modelled as a stochastic process through an expression noise (see next section). We denote by *z*_*x*,*k*,1_(*i*) and *z*_*x*,*k*,2_(*i*) the two alleles at a given locus 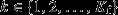 coding for trait 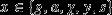 and define them as two integers lying between 1 and *L*_f_. For simplicity, loci coding for the same trait has the same number of possible alleles. To translate these integers into two allelic values *A*_*x*,*k*,1_(*i*) and *A*_*x*,*k*,2_(*i*), we assume an initial minimum and maximum genotypic value, *A*_*x*,min_ and *A*_*x*,max_, respectively, for each trait x in the population (Table2). Allelic values are then defined as

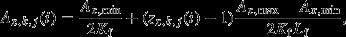
(1a)

for 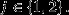 The genotypic value *A*_*x*_(*i*) of trait *x* is then given by the sum of allelic values across loci

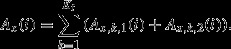
(1b)

#### Phenotypic expression of life-history traits

At birth, an individual's expressed traits (*g*, *α*, *χ*, *y*, and *s*) deviate from their genotypic values, reflecting micro-environmental variation and nonadditive genetic effects (dominance and epistasis). This variation is described by an expression noise *ɛ*_*x*_ with 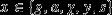 that acts multiplicatively on the genotypic value. Any trait value *x* for individual *i* is then obtained from the following generic equation,

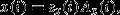
(2a) where *ɛ*_*x*_(*i*) is randomly drawn, once per lifetime, from a normal distribution with mean 1 and a standard deviation specific to each trait *x* (Table2).

Besides expression noise, the juvenile growth rate *g*(*i,t*) is also affected by population-level density dependence,

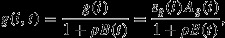
(2b) where *B*(*t*) is the population biomass in year *t* and 1/ρ is the total population biomass at which density dependence halves the juvenile growth rate.

### Life-history processes

An individual *i* in year *t* is characterized by its five life-history traits (*g*(*i,t*), α(*i*), χ(*i*), *y*(*i*) and *s*(*i*)); its age *a*(*i,t*), its length ℓ(*i*,*t*) and its age at maturation *a*_m_(*i*). Together, these determine the four annual life-history processes: somatic and gonadic growth, maturation, reproduction and mortality.

#### Energy allocation to somatic and gonadic growth

Energy allocation between growth and reproduction is described following Quince et al.'s (2008) biphasic seasonal growth model. An individual's net energy acquisition rate, that is the energy surplus after accounting for maintenance, is assumed to scale with its somatic weight *w*(*i,t*) as *w*^*β*^(*i*,*t*), where *β* denotes the production exponent. Juveniles allocate all energy available to somatic growth, whereas adults start by allocating all energy to somatic growth and switch to allocating all energy to gonadic growth after a fraction *p*(*i,t*) of the productive season. *p*(*i,t*) is given by

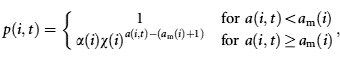
(3a) where *α*(*i*) and *χ*(*i*) lie between 0 and 1 (Quince et al. 2008). A newly matured individual allocates a proportion *α*(*i*) of the productive season to somatic growth; this proportion then decreases geometrically with an annual ratio *χ*(*i*) as the individual ages. This is consistent with von Bertalanffy adult growth.

Assuming a production exponent *β* of 2/3 (Kozlowski and Wiegert 1986; Kozłowski and Wiegert 1987; Lester et al. 2004) and that somatic weight scales with body length according to 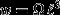, an individual's length-at-age trajectory is given by

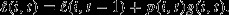
(3b)

Before maturation, the gonad weight *G*(*i,t*) equals 0; after maturation, it is a function of body length at the end of each productive season,

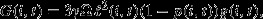
(3c) where *γ* is the ratio of the wet-weight energy density of somatic tissue to gonad tissue. The gonado-somatic index (GSI) is defined as the ratio between gonadic weight and somatic weight, *Γ*(*i*,*t*) = *G*(*i*,*t*)/*w*(*i*,*t*).

#### Maturation

We model sexual maturation using probabilistic maturation reaction norms (PMRNs; Heino et al. 2002b). PMRNs describe the probability that an immature individual matures in dependence on its age and size. We assume a linear PMRN characterized by its intercept *y* and slope *s*. The maturation probability is then given by the logistic equation

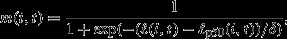
(4a) where ℓ_p50_(*i*,*t*) = *y*(*i*) + *s*(*i*)*a*(*i*,*t*) is individual *i* 's length at 50% maturation probability. The parameter *δ* is determined by the PMRN envelope width *ω*,

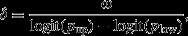
(4b) where logit(*p*) = ln (*p*/(1 – *p*)), and *p*_up_ and *p*_low_ are the upper and lower probability bounds, respectively, for which the PMRN envelope width is defined.

Maturation is modelled as a stochastic process of Bernoulli trials, taking place if a number randomly drawn from a uniform distribution between 0 and 1 is smaller than *m*(*i,t*).

#### Reproduction

An individual's fecundity is defined by dividing its gonad weight by an egg weight, *Q*(*i*,*t*) = *G*(*i*,*t*)/*w*_egg_. From this, the total number of newborns in each year, *N*_0_, is determined by a Beverton–Holt recruitment function (Beverton and Holt 1994),

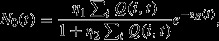
(5) which depends on the population fecundity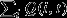 and a lognormally distributed population-level interannual noise factor 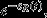 where *ɛ*_*R*_(*t*) is normally distributed (Table2), which represents the influence of environmental fluctuations (e.g. temperature or larval food supply) on recruitment. The parameter *η*_1_ specifies the survival probability of recruits when population fecundity is low and 1/*η*_2_ is the population fecundity at which density dependence halves recruitment, resulting in a maximum asymptotic number of *η*_1_/*η*_2_ recruits.

So far, we have only defined the number of newborns in each year. Each newborn is assigned two parents, selected in proportion to their gonad size through a stochastic process of Bernoulli trials. The adult having the largest gonad weight is determined in each year. Then, an individual is randomly drawn (with replacement) and a number is randomly drawn from a uniform distribution between 0 and this maximal gonad weight. If this number is less than the drawn individual's gonad weight, this individual will be selected as a parent. Hence, the higher an individual's fecundity, the more likely it is to become a parent. The random draws continue until enough parents have been selected. Individuals selected as parents are then randomly combined into pairs for each recruit. Therefore, on average, each individual *i* has a number of offspring in year *t* that is a fraction of the population's recruitment *N*_0_(*t*), equalling the proportion 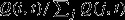 of population fecundity contributed by that individual. After two parents are selected for a newborn, this offspring receives, for each diploid locus, one allele from each parent, carried by two randomly selected parental gametes.

#### Natural and fishing mortality

Three different sources of natural mortality are considered. First, a constant mortality *d*_0_ originates from diseases, senescence, or any stochastic source of mortality unrelated to body size. Second, size-dependent predation generates mortality decreasing with body size according to

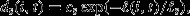
(6a)

where *c*_s_ is the maximum instantaneous predation mortality rate and *ℓ*_s_ is the size at which the predation mortality is decreased by the factor 1/*e*. Third, a growth-dependent mortality,

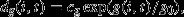
(6b) with *c*_g_ denoting the minimum instantaneous growth-dependent mortality rate and *g*_0_ the growth rate at which the growth-dependent mortality is increased by the factor *e*, accounts for the trade-off between growth and survival. Such a trade-off can originate physiologically when faster growth is achieved at the expense of less energy being available for maintenance (Ernande et al. 2003) or ecologically when the higher energy intake required for faster growth is achieved through more active foraging and thus at the expense of a higher exposure to predation (Abrams 1991; Werner and Anholt 1993; Walters and Korman 1999).

Individuals may also experience size-dependent fishing mortality. The fishery considered is composed of trawlers, characterized by a sigmoid size-selectivity curve,

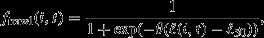
(7a)

where *θ* specifies the steepness of the sigmoid and *ℓ*_50_ is the body size at which the harvest rate equals 50% of its maximum. The instantaneous fishing mortality rate depends on this selectivity curve and on the maximum instantaneous harvest rate *H*,

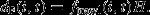
(7b) The total instantaneous mortality rate is then given by

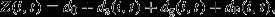
(8) so the probability that individual *i* dies during 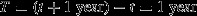 equals *D*(*i*,*t*) = 1 − exp (− *Z*(*i*,*t*)*T*). As for maturation and reproduction, individual mortality is modelled as a stochastic process of Bernoulli trials and occurs if a number randomly drawn from a uniform distribution between 0 and 1 is lower than *D*(*i,t*).

### Measures of population genetic diversity

We follow the effect of fishing on neutral and functional genetic diversity using three genetic diversity indices.

#### Effective population size

We borrow empirical methods of population genetics and estimate effective population size *N*_e_ using the temporal method (Waples 1989; Waples and Yokota 2007). Considering the standardized measure of variance in allele frequency change *F* between two samples at different time points,

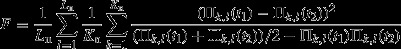
(9a)

where Π_*k*,*l*_(*t*) is the frequency of allelic state *l* at locus *k* at time *t*, and *t*_1_ and *t*_2_ are two sampling years, the effective population size is then obtained as

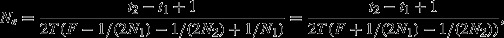
(9b)

where *T* is the generation time in years, and *N*_1_ and *N*_2_ are the population sizes at times *t*_1_ and *t*_2_, respectively (Nei and Tajima 1981; Waples 1989). Denoting by *λ*_*a*_ the fraction of a cohort surviving to age a and by *b*_a_ the mean number of offspring produced in one time interval by individuals aged *a*, *T* is given by 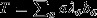. We calculate *F* using neutral markers of the whole population every 20 years and then estimate effective population size *N*_e_ for each time interval. We chose an estimation interval of 20 years, because the bias in *N*_e_ estimates due to overlapping generations decreases with the number of generations in the estimation interval (Waples and Yokota 2007).

#### Additive genetic variance and heritability of quantitative traits

Knowing the genotypic value *A*_*x*_(*i*) of any trait *x*(*i*) for each individual *i*, the population's mean genotypic value 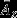 and additive genetic variance *V*_A_(*x*) are directly calculated from the population composition in each year, and the heritability *h*^2^(*x*) is obtained as

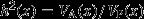
(10) (see, e.g. Lynch and Walsh 1998).

#### Expected heterozygosity

To assess the relative contributions of genetic drift and selection imposed by fishing on the evolution of life-history traits, we estimate the expected heterozygosity over neutral loci on the one hand (eqn 11a) and over functional loci coding for each life-history trait on the other hand (eqn 11b) (Nei and Roychoudhury 1974),

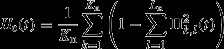
(11a)

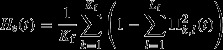
(11b) where Π_*k*,*l*_(*t*) is the frequency of allelic state *l* at locus *k* at time *t*.

Expected heterozygosity will decrease as variability in allele frequencies, and thus genetic diversity, decreases owing to genetic drift and/or selection. Both evolutionary forces affect functional diversity, while neutral heterozygosity depends only on genetic drift, and thus constitutes a baseline allowing detecting the effects of selection whenever functional heterozygosity differs from this baseline.

### Selection differentials

To assess the strength of selection pressures, selection differentials *S*(*x*) are estimated in each year by inverting the breeder's equation *R*(*x*) = *h*^2^(*x*)*S*(*x*), where heritability *h*^2^(*x*) is estimated as described above and the selection response *R*(*x*) is computed as the difference in mean trait value between the new cohort and the population that produced it. Mean-standardized selection differentials are also calculated, by multiplying selection differentials with the trait's mean and dividing by its variance (Matsumura et al. 2012).

### Model parameterization, initial values and runs

We model a population resembling North Sea cod (*Gadus morhua*) with parameters taken from the literature or, when not available, fixed to values yielding plausible emergent properties and patterns; for more details, see Table2.

The initial population is comprised of 220 000 juveniles. For each individual, alleles at each neutral and functional locus are randomly drawn from a uniform distribution between 1 and 10 (i.e. the number of allelic states per neutral and functional locus). Juveniles are initially given a random age between 2 and 6 years old, to avoid the high mortality at younger ages (which, without reproduction, would steeply reduce population size). For each juvenile *i*, initial body size is determined by its genotypic juvenile growth rate, i.e. *g*(*i*,0) = *A*_*g*_(*i*), and its randomly attributed age *a*(*i*,0), while neglecting density dependence and expression noise, so that *ℓ*(*i*,0) = *ℓ*_0_ + *A*_*g*_(*i*)*a*(*i*,0).

We first let the population reach a demographic and evolutionary equilibrium during 17 000 years without fishing. We display all results from this time onward. Model runs start without fishing for 100 extra years. Harvesting then begins and lasts another 100 years. The maximum instantaneous fishing rate *H* takes values from 0.2 to 1 (Table2). Finally, we stop harvesting and explore the genetic trait dynamics after fishing, for 200 more years. All results presented are averages of 25 replicate model runs, carried out with different random seeds.

## Results

### Effects of fishing on life-history trait means

**Figure 1:**
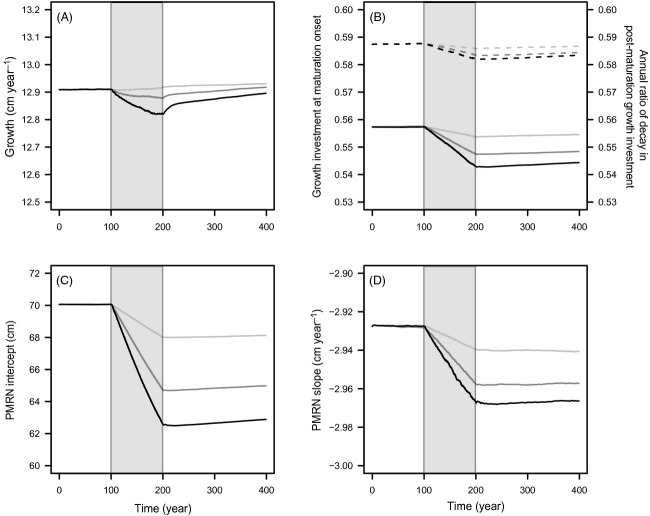
Dynamics of the mean genotypic values of life-history traits before, during and after harvesting. Harvesting (grey shading) starts at *t* = 100 year and stops at *t* = 200 year. Dynamics are shown for three different maximum instantaneous harvest rates: *H* = 0.2 year^−1^ (light grey curves), *H* = 0.6 year^−1^ (dark grey curves) and *H* = 1 year^−1^ (black curves). (A) Juvenile growth rate g. (B) Energy allocation to growth after maturation: growth investment *α* at maturation onset (continuous curve) and annual ratio *χ* of decay in postmaturation growth investment (dashed curve). (C) PMRN intercept *y*. (D) PMRN slope *s*.

As harvesting occurs, the mean genotypic values of all five life-history traits decrease – the higher the harvest intensity, the stronger these effects (Fig.1A–D). When fishing stops, the mean genotypic value of juvenile growth rate almost recovers, for all considered fishing intensities, within the next 200 years, while all other genetic values remain low, despite some very shallow upward trends mostly noticeable for the highest fishing intensity. The phenotypic dynamics of emergent life-history traits (age and size at maturation, length-at-age, and GSI-at-age) follow from the dynamics of the five genetically coded traits in combination with the demographic effects of fishing (Figs S1 and S2).

### Irreversibility of life-history trait evolution after fishing: possible causes

#### Genetic drift due to demographic effects

**Figure 2:**
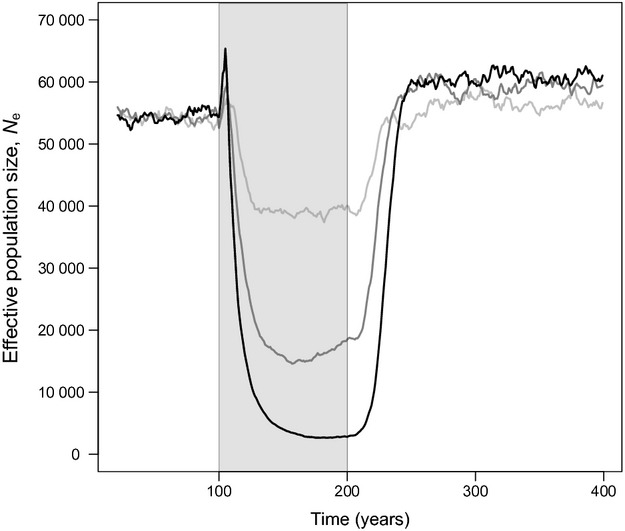
Dynamics of effective population size *N*_e_, before, during and after harvesting. Harvesting (grey shading) starts at *t* = 100 year and stops at *t* = 200 year. Dynamics are shown for three different maximum instantaneous harvest rates: *H* = 0.2 year^−1^ (light grey curves), *H* = 0.6 year^−1^ (dark grey curves) and *H* = 1 year^−1^ (black curves). The effective population size *N*_e_ is shown as a function of time measured in years, and not in generations, because the population's generation time changes throughout the modelled period, equalling, on average, 12.1 year before fishing, 9.9, 8.0 and 7.5 year during fishing, and 11.9, 11.6 and 11.4 year after fishing (at *H* = 0.2 year^−1^, *H* = 0.6 year^−1^, and *H* = 1 year^−1^, respectively).

Fishing modifies effective population size and thus changes the rate of genetic drift affecting allelic frequencies (Fig.2). As harvesting starts, effective population size first increases and then steeply drops, both occurring with larger amplitudes when fishing intensity is high. After fishing is stopped, effective population size recovers to levels equal or slightly higher than the initial ones, but with a time lag that increases when fishing intensity is stronger (around 30 years at *H* = 0.2 year^−1^, 40 years at *H* = 0.6 year^−1^ and 50 years at *H* = 1 year^−1^). Effective population sizes after recovery are larger when fishing intensities are higher (Fig.2), mirroring trends in population size (Figs S2 and S3). This is because genetic adaptations during fishing drive individuals towards higher fecundity and earlier maturation, which raises the number and lifetime fecundity of mature individuals, and thus increases recruitment and population size.

#### Selection differentials

**Figure 3:**
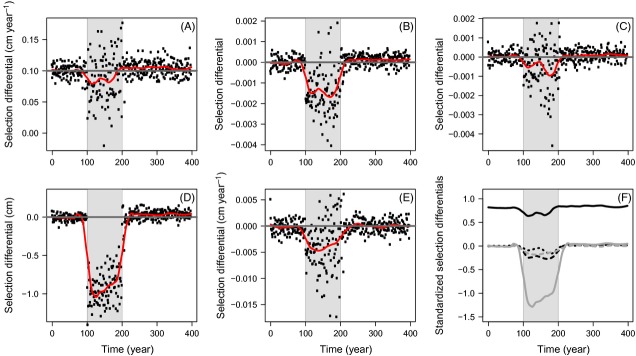
Selection differentials (A–E) and mean-standardized selection differentials (F) of life-history traits before, during, and after harvesting. Harvesting (grey shading) starts at *t* = 100 year and stops at *t* = 200 year. Selection differentials are shown for a maximum instantaneous harvest rate of *H* = 1 year^−1^. In (A–E), the red line is a smoothing function (loess with a span of 0.2) and horizontal grey lines give the baseline of 0 selection differential for all traits except for juvenile growth rate (baseline of 0.1). (A) Juvenile growth rate g (continuous black line in F). (B) Growth investment *α* at maturation onset (dashed black line in F). (C) Annual ratio *χ* of decay in postmaturation growth investment (dotted black line in F). (D) PMRN intercept y (continuous grey line in F). (E) PMRN slope s (dashed grey line in F). (F) Mean-standardized selection differentials of the five life-history traits, shown by smoothing functions (loess with a span of 0.2).

Before fishing, the population is at an evolutionary equilibrium and no selection differential is statistically different from 0 at the 1% risk level (*α*: *t*(98) = −0.32, *P* = 0.74; *χ*: *t*(98) = 0.83, *P* = 0.41; *y*: *t*(98) = −2.21, *P* = 0.03; *s*: *t*(98) = −1.5, *P* = 0.14), except for growth selection differentials (*g*: *t*(98) = 98.1, *P* < 2.2 × 10^−16^), which are positive with a mean of 0.1 (Fig.3). The latter, however, do not reflect selection, but just the trade-off between growth and survival: because of this trade-off, slow-growing phenotypes are overrepresented in older age classes (similar to ‘Lee's phenomenon’; Lee 1912), so that the difference in mean growth between a new cohort and the whole population is positive.

During harvesting, selection differentials of all traits decrease, with the differences to preharvest values being statistically significant at the 1% risk level for all traits (*g*: *t*(99) = −3.46, *P* < 0.001; *α*: *t*(99) = −13.41, *P* < 2.2 × 10^−16^; *χ*: *t*(99) = −5.3, *P* < 10^−6^; *y*: *t*(99) = −38.9, *P* < 2.2 × 10^−16^; *s*: *t*(99) = −8.1, *P* < 10^−11^). The initial decrease is followed by an increase for the PRMN intercept and slope (red lines in Fig.3D,E), and by an initial increase and a subsequent drop for the other traits (red lines in Fig.3A–C).

After fishing, selection differentials increase and slightly exceed preharvest values for all traits (Fig.3A–D) except the PMRN slope (Fig.3E). Even though they are statistically higher than their preharvest values at the 1% risk level for juvenile growth rate (*g*: *t*(99) = 3.04, *P* = 0.003; Fig.3A) and growth investment at maturation onset (*α*: *t*(99) = 2.6, *P* = 0.01; Fig.3B), selection differentials remain very low.

Mean-standardized selection differentials (Fig.3F), which allow comparing selection strength across traits, undergo reductions of similar amplitude across all traits – except for the PMRN intercept, for which negative selection is much stronger.

#### Effects of fishing on genetic and phenotypic variances of life-history traits

**Figure 4:**
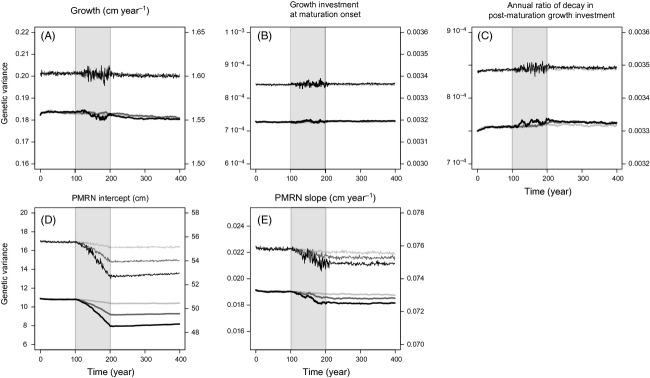
Evolutionary dynamics of genetic variances (thick lines, left vertical axes) and phenotypic variances (thin lines, right vertical axes) of life-history traits before, during and after harvesting. Harvesting (grey shading) starts at *t* = 100 year and stops at *t* = 200 year. Results are shown for three different maximum instantaneous harvest rates: *H* = 0.2 year^−1^ (light grey curves), *H* = 0.6 year^−1^ (dark grey curves) and *H* = 1 year^−1^ (black curves). (A) Juvenile growth rate g. (B) Growth investment *α* at maturation onset. (C) Annual ratio *χ* of decay in postmaturation growth investment. (D) PMRN intercept y. (E) PMRN slope *s*.

Before harvesting, the dynamics of additive genetic and phenotypic variances of all traits are steady, indicating the absence of selection (Fig.4A–E). Heritability equals 0.11 for growth, 0.22 for growth investment at maturation onset and its subsequent annual ratio of decay, 0.18 for the PMRN intercept, and 0.25 for the PMRN slope.

As fishing starts, the dynamics of both functional genetic and phenotypic diversity are affected. First, fluctuations in genetic and/or phenotypic variances are amplified, with larger amplitudes of change in the latter. Second, high fishing pressure induces a reduction in the genetic and phenotypic variances of several traits. Most noticeable is the PMRN intercept, with a decrease of roughly 3 cm^2^ in genetic variance for the highest fishing mortality (Fig.4D). Genetic variance decreases to a lesser degree for growth (Fig.4A) and the PMRN slope (Fig.4E) and slightly increases for the annual ratio of decay in postmaturation growth investment (Fig.4C). Most variances that are reduced by fishing do not recover to previous levels after fishing, although shallow upward trends can be noticed for the PMRN intercept.

### Relative contributions of genetic drift and selection to losses in functional genetic variability

**Figure 5:**
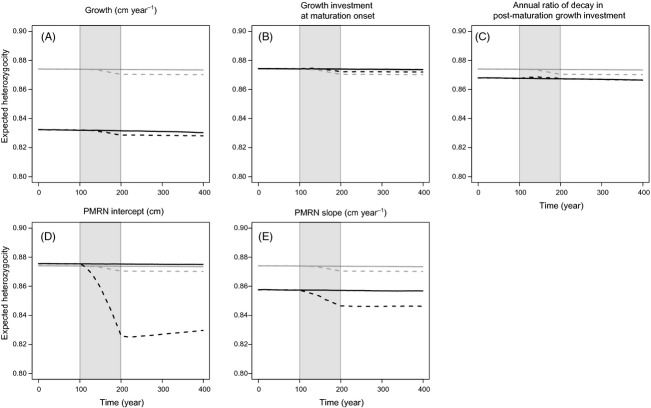
Comparison of temporal trends in neutral and functional genetic diversity before, during and after harvesting. Harvesting (grey shading) starts at *t* = 100 year and stops at *t* = 200 year. Expected heterozygosity *H*_e_ of neutral loci (grey lines) and functional loci (black lines) are compared for the five life-history traits: (A) juvenile growth rate *g*, (B) growth investment *α* at maturation onset, (C) annual ratio χ of decay in postmaturation growth investment, (D) PMRN intercept *y* and (E) PMRN slope *s*. Results are shown for maximum instantaneous harvest rates that are moderate (*H* = 0.4 year^−1^; continuous lines) or strong (*H* = 1 year^−1^; dashed lines).

At the beginning of model runs, neutral heterozygosity is slightly higher than functional heterozygosity (Fig.5), because stabilizing selection acts on functional loci during the initialization period meant to reach evolutionary equilibrium (17 000 years), eroding part of the functional genetic diversity. As a consequence, the amplitudes of changes in neutral and functional heterozygosity are to be compared, rather than their absolute values, to distinguish the impacts of neutral and adaptive evolution on functional genetic diversity.

As expected from the results for effective population size, neutral heterozygosity is constant under moderate fishing mortality (continuous grey lines in Fig.5), suggesting weak fisheries-induced genetic drift, whereas it diminishes under high fishing mortality (dashed grey lines in Fig.5) due to increased genetic drift. Notice, however, that the decrease amounts to <1% of the initial value, which is expected given the range of effective populations sizes (3000–40 000, Fig.2), and the fact that heterozygosity decreases by the ratio 1 − 1/(2*N*_e_) per generation under genetic drift (Wright 1931). At high fishing intensity, a much more pronounced decrease in functional heterozygosity is observed for the PMRN intercept (≈6% Fig.5D) and PMRN slope (≈2%, Fig.5E). In contrast, functional heterozygosity decreases at a rate similar to neutral heterozygosity for juvenile growth (Fig.5A), decreases at a lower rate for growth investment at maturation onset (Fig.5B) or stays approximately constant for the annual ratio of decay in postmaturation growth investment (Fig.5C).

However, as the rate of change in genetic diversity is expected to be influenced by its own initial value, whether evolution is neutral (Nei et al. 1975) or adaptive (Lande 1980; Lande and Arnold 1983), the differences between neutral and functional heterozygosity levels at the beginning of model runs preclude any conclusive interpretation of slight differences in changes when fishing occurs, especially when changes occur at a scale of only a few percentage or less. This holds particularly for the traits with the strongest initial differences, that is for juvenile growth rate, the annual ratio of decay in growth investment and the PMRN slope (Fig5A,C,E). Therefore, one can firmly conclude that both genetic drift and selection are contributing to diminishing functional genetic diversity only for the PMRN intercept (Fig.5D), with their respective contributions being unclear for growth (Fig.5A) and the PMRN slope (Fig.5E). For the two traits involved in growth investment after maturation (Fig.5B,C), selection seems to counteract the effect of genetic drift, but the amount of change in neutral and functional heterozygosity (<1%) can be deemed negligible. Most importantly, whatever the source of loss in genetic diversity and its amplitude, there is little recovery, if any, after fishing stops, except for the PMRN intercept under the strongest fishing intensity (black-dashed line in Fig.5D).

## Discussion

### Fisheries-induced adaptive evolution and its reversal: comparison with previous models

Eco-genetic individual-based models have been recently used to explore the eco-evolutionary dynamics of harvested fish populations (e.g. Baskett et al. 2005; Dunlop et al. 2007, 2009a,b; Thériault et al. 2008; Enberg et al. 2009). Our model belongs to this modelling framework with the main novelty lying in an explicit description of the population genetics of life-history traits, using a finite number of loci and alleles coding for each trait, together with the gametic transmission of alleles (see Wang and Höök 2009; Kuparinen and Hutchings 2012 for earlier approaches of this kind). In contrast, most previous models relied on a quantitative genetic modelling approach, which assumes that life-history traits are influenced by an infinite number of loci, each of small effect (Huisman and Tufto 2012).

Three observations are noteworthy in comparison with those previous studies. First, our model makes consistent predictions for the evolution of the mean genotypic values of life-history traits under fishing pressure, namely reduced juvenile growth, increased reproductive investment and earlier maturation through a reduced PMRN intercept. Second, the reversal of genetic adaptations during a fishing moratorium was faster in previous eco-genetic studies than in our model. Therefore, weak selection differentials cannot be the only cause of the slow recovery of trait genotypic values to their initial levels. Instead, our model has revealed a fisheries-induced erosion of adaptive potential that is hampering this recovery. This erosion went unnoticed in eco-genetic models based on quantitative genetic principles, in which the loss of genetic variation is not observed. Most probably, this is because, unlike multilocus model such as ours, those models assumed a constant genetic variance under linkage equilibrium (Huisman and Tufto 2012). Third, we find that phenotypic values of emergent life-history traits – age and size at maturation, length-at-age and GSI-at-age – partly recover thanks to phenotypic compensation (Fig. S1). Such partial phenotypic recovery also explains, at least partly, the lack of genetic recovery: it reduces the gap between the expressed phenotype and the new phenotypic trait values favoured when fishing stops and hence lowers the selection pressures towards the initial genotypic trait values.

### Processes affecting evolutionary recovery

We have examined the influence of three nonmutually exclusive processes that can hamper the reversal of fisheries-induced evolution: increased rate of genetic drift, low strength of selection and reduced additive genetic variance.

#### Effective population size

Our results indicate that fishing may decrease effective population size, thus increasing the rate of neutral evolution due to genetic drift. This decrease is in agreement with some empirical genetic studies on exploited fish population (Smith et al. 1991; Hauser et al. 2002; Turner et al. 2002; Hutchinson et al. 2003; Hoarau et al. 2005; Pinsky and Palumbi 2014; but see Ruzzante et al. 2001; Therkildsen et al. 2010). Such a decrease could limit a population's adaptive response to selection, because genetic drift in small populations decreases the chance of fixation of beneficial alleles, which can counteract the effects of selection. This, however, may only happen when effective population sizes reach values of a few tens, which are not observed in our model (Robertson 1960). The balancing of selection by genetic drift therefore mostly applies to small populations, for example of marine coral reef fish or freshwater species. In addition, our model shows that when fishing ceases, effective population size bounces back and surpasses its prefishing level. This suggests that the effect of fishing on the rate of genetic drift does not last long during a subsequent moratorium. We therefore expect that for most large marine fish populations, as in our model, the rate of genetic drift will not counteract the reversal of fisheries-induced evolution.

A strong decrease in effective population size due to fishing raises concerns about potential losses of genetic variability due to genetic drift. Such losses imply a risk of inbreeding, which may in turn increase extinction risks (reviewed in Frankham 2005). However, our results suggest that, whatever the fishing intensity considered, genetic diversity (expected heterozygosity) remains almost unaltered by genetic drift in large exploited marine fish populations, as expected from the range of *N*_e_ values observed in our model (Wright 1931). Although the loss of genetic variability may thus appear to be a secondary issue, empirical studies of *N*_e_ dynamics, using DNA from archived otoliths or scales (Poulsen et al. 2006; Nielsen and Hansen 2008), could still enable a ‘retrospective monitoring’ of other aspects of conservation and management interest, such as inferring the historical demography of exploited fish stocks from the link between *N*_e_ and population abundance (Fig. S2). Reconstructing demographic history would enable integrating baseline estimates, in terms of preharvest parameters, as reference points in fisheries management.

In our model, the ratio *N*_e_/*N* of effective population size to population census size equals approximately 0.1 on average (Fig. S3), which agrees with empirical evidence in general (Frankham 1995; Palstra and Ruzzante 2008), although empirical estimates of *N*_e_/*N* suffer from uncertainty (Palstra and Fraser 2012). However, studies on marine fishes have also documented extremely low ratios *N*_e_/*N*, around 10^−2^ to 10^−6^ (Smith et al. 1991; Hauser et al. 2002; Turner et al. 2002; Hoarau et al. 2005). Such large discrepancies between *N*_e_ and *N* in marine fish species have been attributed mainly to high interindividual variability in reproductive success. This reproductive skew can arise from (i) the influence of environmental variability on recruitment (which, combined with large fecundity, may lead to ‘sweepstake recruitment’ events; Hedgecock 1994) and/or from (ii) productivity differences among isolated subpopulations (Turner et al. 2002). Both mechanisms are not accounted for in our model, which could explain why we observe a larger *N*_e_/*N* ratio than some empirical studies. How this affects the interpretation of our results is an interesting question: for a much lower *N*_e_/*N* ratio, it is theoretically possible that fishing only hits the ‘noneffective’ part of a population, while sparing its effective part. In practice, however, it is well known that, in most fishes, the most successful spawners are the older and larger ones, which typically also are most vulnerable to fishing. We therefore think that all ecological mechanisms potentially further reducing *N*_e_ would rather increase the risk of inbreeding due to the fisheries-induced decrease in effective population size observed in our model.

#### Selection differentials and additive genetic variance

Our study highlights that the pace of reversal of fisheries-induced life-history evolution after the cessation of fishing is hampered by two processes: small natural selection differentials compared to those imposed by fishing and a reduction in the genetic variability of traits, as shown by the decrease in their additive genetic variance. While the former effect had already been pointed out in previous eco-genetic studies analysing fisheries-induced evolution (Enberg et al. 2009; Kuparinen and Hutchings 2012), the latter effect so far has not received any attention within this framework. At the timescale considered in our study, the creation of new functional alleles through mutation is a rare event, and thus neglected, so standing quantitative genetic variation is the main determinant of the modelled population's ability to evolve. This genetic variation can be eliminated at a fast rate through selection and genetic drift, raising concerns for a population's adaptability (Ryman et al. 1995).

While the considered multilocus genetic architecture of life-history traits takes our model an important step closer towards the genetic complexity of real populations, leaving out the effects of dominance and epistasis remains a simplifying assumption. Overcoming this simplification in future research would be desirable, as the nonadditive genetic variance enabled by dominance and epistasis can represent a large proportion of a population's total genetic variance in traits associated with reproductive fitness (Heath et al. 1994; Pante et al. 2002; but see Nilsson 1992). Also, nonadditive genetic variance may be converted into additive genetic variance during processes of severe population reduction, thus increasing additive genetic variance instead of decreasing it (e.g. Bryant et al. 1986; Fernández et al. 1995). However, this effect is expected to be a short-termed (Frankham 2005), and it is unknown whether it is widespread or not.

### Fishing reduces functional genetic variation

In our results, losses of genetic variability due to pure genetic drift (neutral loci) are extremely weak, which is as expected at the observed range of effective population sizes: given that heterozygosity decreases by the factor 1−1/2*N*_e_ per generation under pure genetic drift, 100 generations (i.e. more than 700 years in our model) are required to reduce it by 2% when *N*_e_ = 3000, which is the lowest *N*_e_ value observed in our model under strong fishing intensity. It is therefore unlikely that in large marine fish populations, fisheries-induced genetic drift significantly reduces functional genetic variation.

We also find that differences between the temporal trends of neutral and functional expected heterozygosity increase when fisheries-induced changes in the mean genotypic values of life-history traits are larger: the loss in genetic variability at neutral and functional loci is almost equal for most traits, except for the PMRN intercept. Therefore, strong stabilizing selection obviously occurs for the latter, whereas for the other four traits, losses in functional genetic diversity remain very small and equivalent to losses in neutral genetic diversity.

It also interesting to consider whether the observed fisheries-induced evolutionary responses of mean genotypic life-history trait values are due to selective pressures or genetic drift or a mixture of both. As the observed directions of these evolutionary responses are adaptive (although they might become maladaptive after the cessation of fishing), they are most likely due to fisheries-induced selection. Moreover, genetic drift modifies allelic frequencies randomly, thus equally leading to beneficial or detrimental changes in individual fitness, which would tend to counteract the effects of selection. This further supports the interpretation that the evolutionary dynamics of at least the mean genotypic values of life-history traits are due to fisheries-induced selection, in agreement with new experimental results using guppies as model species (Van Wijk et al. 2013).

Besides these considerations, the co-variation observed between heterozygosity of neutral and functional loci suggest that the temporal dynamics of neutral genetic diversity might possibly be a useful surrogate of the temporal dynamics of functional genetic diversity (Merilä and Crnokrak 2001; Leinonen et al. 2007). As yet, however, this conjecture needs to be treated with some caution, considering the current lack of empirical evidence and the fact that most changes in genetic diversity observed in our model were relatively weak, except for the PMRN intercept.

Two main results of our study are that the observed losses in additive genetic variance and functional allelic diversity are permanent, prevailing even after fishing is stopped, and that the resulting loss in evolutionary potential impedes the genetic recovery of life-history traits. Long-term selection has been shown to deplete additive genetic variance in animal models such as *Drosophila melanogaster* (e.g. Robertson 1955), but not always, in particular when population size was reasonably large (e.g. Yoo 1980). We neglected mutations and nonadditive genetic effects in our model, which could replenish additive genetic variance and allow initial mean genotypic values to be restored. However, mutation rates of functional DNA are so low (10^−9^ per locus per generation; Li 1997) that they are irrelevant at the timescale considered (although this rate could in theory increase under environmental stress; Lamb et al. 1998).

### Management implications

Our results show the importance of managing genetic impacts of fishing on exploited populations. First, fisheries-induced evolution may be detrimental for a stock's productivity, as population biomass does not fully recover to its prefishing level after fishing is stopped (Fig. S2). This is a concern for present and future profits of the fishing industry, as our model suggests that genetic adaptations induced by fishing do not recover even within 200 years without fishing.

Mitigating fisheries-induced genetic drift is important for reducing the potential risk of inbreeding and its impact on evolutionary potential. Although conflicting empirical results emerge from different estimates of effective population size (but see meta-analysis by Pinsky and Palumbi 2014), maintaining its level appears critical. To do so, genetic monitoring of neutral molecular markers, or alternatively, under certain circumstances, population demographic properties can be used to infer effective population size (Waples and Yokota 2007). In large populations, estimates of effective population size should be treated with caution, as the genetic-drift signal becomes small, and this may lead to low estimation precision (Hare et al. 2011). In our analyses, we did not test whether the decrease in effective population size due to fishing occurs only due to demographic effects or also due to the selective effects of fishing (e.g. through an increase in the variance of reproductive fitness among individuals). Our model could be used to carry out such a test as part of future research, by comparing estimates of effective population size when the modelled population is evolving to estimates when evolution is artificially precluded.

Regarding the reduction of functional diversity due to fisheries-induced adaptive evolution, our findings for the PMRN intercept highlight the importance of mitigating selection on maturation imposed by fishing gears, both to limit changes in mean genotypic and phenotypic values of life-history traits, and to avoid losses of evolutionary potential. A theoretical study on Arctic cod has shown that fishing with a dome-shaped size-selectivity curve could mitigate the evolution of mean age and size at maturation (Jørgensen et al. 2009), but whether this could also avoid significant losses of functional genetic variance remains to be examined.

## Supporting Information

**Figure S1.** Dynamics of the mean phenotypic values of emergent life-history traits before, during, and after harvesting. **Figure S2.** Population demography before, during, and after harvesting. **Figure S3.** Dynamics of the effective population size *N*_e_ before, during, and after harvesting and its co-variation with emergent demographic properties due to fishing.
