# Supplementary material for: Fisheries-induced neutral and adaptive evolution in exploited fish populations and consequences for their adaptive potential
Source: Evol Appl. 2014 Dec 2;8(1):47–63. doi: 10.1111/eva.12220 (PMC4310581; doi:10.1111/eva.12220)
Supplement: Supplementary file 1 [file eva0008-0047-sd1.docx]

## Supporting Information

## 1. Effects of fishing on emergent life-history trait means

In addition to changing the mean genotypic values of all five life-history traits, fishing also changes the means of emergent life-history traits: age and size at maturation, length-at-age, and GSI-at-age (Fig. S1A-D). Naturally, the latter changes can be observed only at the phenotypic level.

Age at maturation decreases during harvesting as a result of three concomitant mechanisms (Fig. S1A). First, the evolutionary decrease of the PMRN intercept (Fig. 1C) increases the probability of younger individuals to mature. Second, the reductions in population size (Fig. S2A) and population biomass (Fig. S2C) relax density dependence. This increases the phenotypic juvenile growth rate (not shown) to the extent of overcompensating the evolutionary decrease of its mean genotypic value, thus allowing individuals to mature earlier. Third, fishing causes a truncation of the population’s age and size structure. Consequently, the population is dominated by young and small individuals, which increases the observed proportion of individuals maturing young and small.

Everything else being equal, increased growth due to the relaxation of density dependence would increase the size at maturation when the PMRN slope is negative, as assumed for our model population (Fig. 1D). Yet, size at maturation strongly diminishes during harvesting, as a consequence of the combined evolution of the PMRN intercept towards smaller sizes (Fig. 1C) and the truncation of the population’s age and size structure (Fig. S2F).

Since individuals at age 3 yr are predominantly juvenile, the mean length at that age increases during harvesting (Fig. S1C): this is because relaxed density dependence increases the phenotypic juvenile growth rate (see above). Conversely, since individuals at age 7 yr are predominantly adult, the mean length at that age decreases during harvesting (Fig. S1C): this is because the trade-off between growth and reproduction is expressed earlier due to earlier maturation (Fig. S1A) and the post-maturation growth investment decreases (Fig. 1B), implying that, everything else being equal, adults tend to invest more into reproduction than into growth than they do without harvesting.

The decrease in adult length, and thus weight, and the increase in reproductive investment raise the GSI at all ages (shown in Fig. S1D for ages 3 yr and 7 yr).

After harvesting has stopped, the emergent life-history traits shown in Fig. S1 recover only partly, though to different degrees. It is interesting to notice, for instance, the seemingly permanent changes in age and size at maturation and in GSI at age 7 yr. In contrast, length at age 3 yr recovers fully, because of the strong density dependence occurring without fishing.


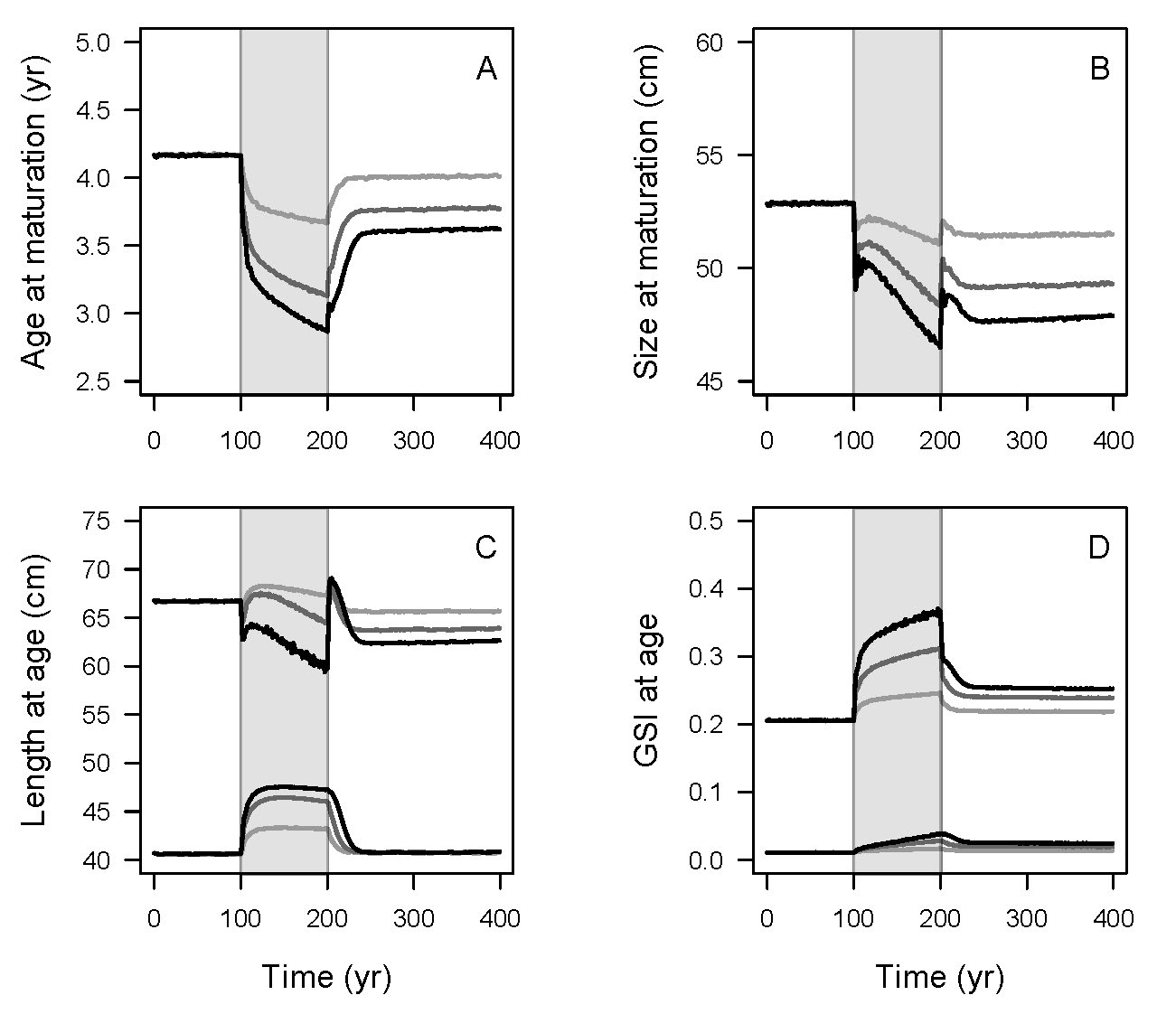
Figure S1. Dynamics of the mean phenotypic values of emergent life-history traits before, during, and after harvesting. Harvesting (grey shading) starts at *t* = 100 yr and stops at *t* = 200 yr. Dynamics are shown for three different maximum instantaneous harvest rates: $H=0.2\text{ yr}^{-1}$ (light-grey curves), $H=0.6\text{ yr}^{-1}$ (dark-grey curves), and $H=1\text{ yr}^{-1}$ (black curves). (A) Age at maturation. (B) Size at maturation. (C) Mean length at age 3 yr (below) and age 7 yr (above). (D) Mean GSI at age 3 yr (below) and age 7 yr (above).

## 2. Effects of fishing on emergent demographic properties

Fishing impacts population demography both directly and through its effects on life-history traits. It reduces population size (Fig. S2A), recruitment (Fig. S2B), population biomass (Fig. S2C), spawning stock biomass (Fig. S2D), population fecundity (Fig. S2E), and the numbers of both mature and immature individuals (Fig. S2F).

For weak to moderate fishing intensity (light-grey and dark-grey curves in Fig. S2), these demographic properties of the stock first drop and then slightly rebound during fishing. This rebound is due to the adaptive changes in life-history traits described above, which partly mitigate the direct demographic effects of fishing (see Enberg et al. 2009 for a similar phenomenon). For high fishing intensity (black curves in Fig. S2), those adaptations are not sufficient to counterbalance the direct demographic effects, so the demographic properties steadily decline without rebound, though more quickly at the beginning of the fishing period, until fishing ceases.

After fishing has stopped, the demographic properties increase to a higher level relative to the initial population (population size, Fig. S2A; recruitment, Fig. S2B; population fecundity, Fig. S2E; and number of mature individuals, Fig. S2F), to an equivalent level (spawning stock biomass, Fig. S2D; number of immature individuals, Fig. S2F), or to a lower level (population biomass, Fig. S2C). This can be understood as follows. Since genetic adaptations during fishing have driven individuals towards being more fecund and maturing earlier, the number of mature individuals and their fecundity have increased, raising recruitment and thus population size. Although adults are more numerous, they are younger and smaller: those changes roughly balance, so spawning stock biomass rebounds to its pre-harvest level. Finally, although recruitment has increased, the larger population size does not compensate for the smaller sizes of adults resulting from earlier maturation, so population biomass remains lower than its pre-harvest level.


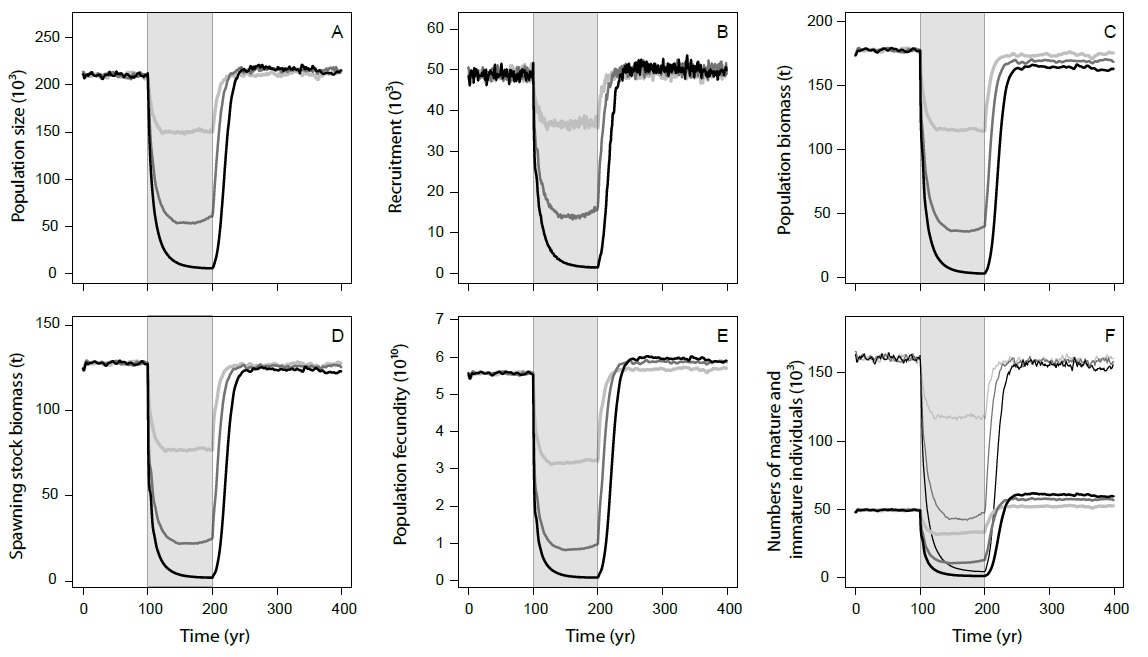


Figure S2. Population demography before, during, and after harvesting. Harvesting (grey shading) starts at *t* = 100 yr and stops at *t* = 200 yr. Dynamics are shown for three different maximum instantaneous harvest rates: $H=0.2\text{ yr}^{-1}$ (light-grey curve), $H=0.6\text{ yr}^{-1}$ (dark-grey curve), and $H=1\text{ yr}^{-1}$ (black curve). (A) Population size *$N$*. (B) Recruitment. (C) Population biomass. (D) Spawning stock biomass. (E) Population fecundity. (F) Numbers of mature individuals (continuous lines) and immature individuals (dashed lines).

## 3. Relationships between effective population size and emergent demographic properties

Effective population size (Fig. S3A) co-varies positively with many demographic properties: its reduction through fishing is associated with a decrease in population size (Fig. S3B), number of mature individuals (Fig. S3C), recruitment (Fig. S3D), spawning stock biomass (Fig. S3E), and population fecundity (Fig. S3G). Its co-variation with mean per capita fecundity is less pronounced, as this merely falls from slightly above 1 without fishing to slightly below 1 under fishing (Fig. S3F).

All aforementioned relations are roughly linear, but have different slopes. For example, the number of mature individuals and recruitment decrease roughly at the same speed as effective population size (slopes of linear regression: 0.94 and 0.91, respectively, with R²=0.99 in both cases; Figs. S3C and S3D), whereas population size decreases about four times faster than effective population size (slope of linear regression: 3.98, with R² = 0.99; Fig. S3B).


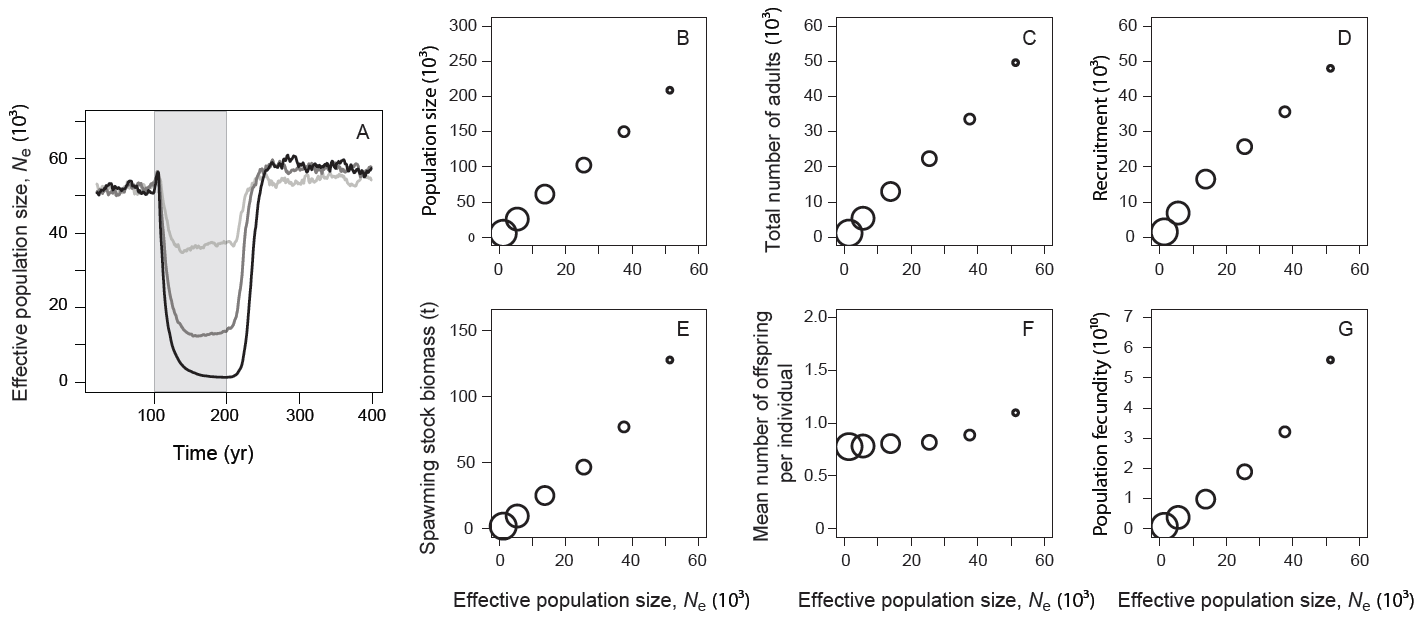


Figure S3. Dynamics of the effective population size *$N_{\text{e}}$* before, during, and after harvesting and its co-variation with emergent demographic properties due to fishing. (A) Harvesting (grey shading) starts at *t* = 100 yr and stops at *t* = 200 yr. Dynamics are shown for three different maximum instantaneous harvest rates: $H=0.2\text{ yr}^{-1}$ (light-grey curves), $H=0.6\text{ yr}^{-1}$ (dark-grey curves), and $H=1\text{ yr}^{-1}$ (black curves). The effective population size *$N_{\text{e}}$* is estimated in time intervals of 20 yr. (B-G) Co-variation of effective population size *$N_{\text{e}}$* after 100 yr of harvesting with six emergent demographic properties, for maximum instantaneous harvest rates $H$ varying from 0 to $\text{1 yr}^{\text{-1}}$ in steps of 0.2: (B) population size *$N$*, (C) number of mature individuals, (D) recruitment, (E) spawning stock biomass, (F) mean per capita fecundity, and (G) population fecundity. The diameter of circles is proportional to the maximum instantaneous harvest rate.
